# Rattlesnakes are extremely fast and variable when striking at kangaroo rats in nature: Three-dimensional high-speed kinematics at night

**DOI:** 10.1038/srep40412

**Published:** 2017-01-13

**Authors:** Timothy E. Higham, Rulon W. Clark, Clint E. Collins, Malachi D. Whitford, Grace A. Freymiller

**Affiliations:** 1 Department of Biology, University of California, Riverside, CA 92521, USA; 2 Department of Biology, San Diego State University, San Diego, CA 92182, USA

## Abstract

Predation plays a central role in the lives of most organisms. Predators must find and subdue prey to survive and reproduce, whereas prey must avoid predators to do the same. The resultant antagonistic coevolution often leads to extreme adaptations in both parties. Few examples capture the imagination like a rapid strike from a venomous snake. However, almost nothing is known about strike performance of viperid snakes under natural conditions. We obtained high-speed (500 fps) three-dimensional video in the field (at night using infrared lights) of Mohave rattlesnakes (*Crotalus scutulatus*) attempting to capture Merriam’s kangaroo rats (*Dipodomys merriami*). Strikes occurred from a range of distances (4.6 to 20.6 cm), and rattlesnake performance was highly variable. Missed capture attempts resulted from both rapid escape maneuvers and poor strike accuracy. Maximum velocity and acceleration of some rattlesnake strikes fell within the range of reported laboratory values, but some far exceeded most observations. Thus, quantifying rapid predator-prey interactions in the wild will propel our understanding of animal performance.

Everything is eaten by something. Thus, predation is a fundamental force in ecology and evolution, structuring both mechanics of movement within an organism and the web of interacting organisms within an ecosystem^1^,^2^. Both predators and prey can evolve extreme adaptations as a result of the coevolutionary “arms race” between them. Predator-prey interactions are, therefore, model systems in studies of behavior, functional morphology, and biomechanics. For example, the fast-start evasion response of fish is a major factor shaping morphology and behavior, and many of studies have examined the mechanics of those responses at a variety of levels^3^,^4^,^5^,^6^,^7^,^8^,^9^.

Predation can be a relatively rare event; it is also unpredictable and often extremely fast. Many predators attempt to maximize their chance of success by ambushing prey with high-speed pursuit or attack behaviors^10^,^11^,^12^,^13^; prey, in turn, have evolved remarkably quick detection and evasion tactics, often initiating a complex, three-dimensional escape within milliseconds^14^,^15^,^16^,^17^. Thus, despite the accuracy of the predatory strike being critical for success^18^,^19^, we know little about the functional basis for variation in strike accuracy in nature.

The animal kingdom is replete with examples of extreme specialization for capturing prey or escaping predators^20^,^21^,^22^,^23^. Rattlesnakes and other vipers, for example, feed by ambushing prey with an extremely rapid envenomating strike^24^,^25^,^26^. Several studies have examined rattlesnake feeding strike behavior in the laboratory under controlled conditions^24^,^27^,^28^,^29^. A recent study compared defensive strike kinematics and performance of two pitvipers to ratsnakes, and found that both harmless and venomous snakes can strike with relatively high accelerations^29^. Although there have been qualitative field observations of rattlesnake strikes^25^,^30^,^31^, it is unclear how the high-speed dynamics of the interactions occur. How fast are rattlesnake strikes in nature, and how are these strikes initiated and executed in relation to a mobile prey? We examined natural predator-prey interactions between the Mohave rattlesnake (*Crotalus scutulatus*) and Merriam’s kangaroo rat (*Dipodomys merriami*). Mohave rattlesnakes specialize on kangaroo rats, incorporating them as the bulk of their diet^32^,^33^. We addressed the following two questions: (1) What is the natural strike performance of *C. scutulatus* and evasive performance of *D. merriami*? We predict that performance of rattlesnakes will be higher in nature compared to laboratory conditions due to the potential for lower stress and natural prey behavior. We predict high acceleration from kangaroo rats in order to evade rapid strikes. (2) What factors determine the success of a strike or an escape? We predict that the evasive maneuvers from kangaroo rats will define the outcome of the interactions (framework presented in Fig. 1).

## Results

**Figure 2:**
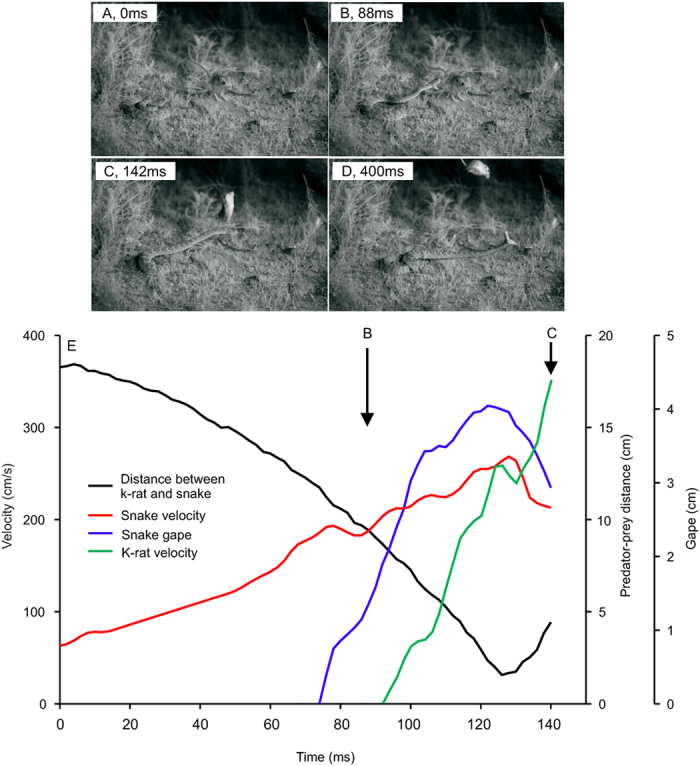
Example sequence from a snake striking and missing due to an evasive maneuver from the kangaroo rat. Photos (A–D) are from the strike outlined in panel E, and photos (B and C) are indicated on the graph. The photos in panels A and D occurred outside of the graph area. Panel D shows a second gape expansion after the initial miss. This was also unsuccessful.

We recorded four successful prey capture attempts and four missed attempts, with average snake body temperatures of 23.1 ± 1.5 °C during the strikes. One missed strike could not be analyzed quantitatively, and so was retained only for general observations. Snake performance was variable and, in several cases, very high. There was a wide range of strike speeds and accelerations (Table 1), and these did not appear linked to capture success. The response time of the kangaroo rats, for those trials in which the kangaroo rat was stationary prior to the snake strike, was 61.5 ± 10.6 ms. Two failed attempts were due to snake error, but two failed attempts were caused by escape maneuvers by the kangaroo rat (Fig. 2; Supplementary Video S1; see videos on YouTube at https://youtu.be/PBEp2LtQwZ8 and https://youtu.be/jCxvIk8wS_8). In these two cases, the time between initiating the escape response and completely clearing the trajectory of the snake was 24 ms and 30 ms. The basis for successful strikes was also variable. For example, one successful strike occurred when the kangaroo rat was stationary and facing away from the snake, likely precluding the use of vision for detecting the snake. However, another successful attempt involved the kangaroo rat facing the snake in a stationary position. Although the kangaroo rat did begin to initiate an escape response, the strike was initiated 4.8 cm from the kangaroo rat and the entire event (from beginning of snake movement until contact) lasted 78 ms. Finally, one hit occurred while the kangaroo rat was in mid air (hopping along a path perpendicular to the orientation of the snake) and the rattlesnake captured it before it could land and escape.

## Discussion

This study is the first to quantify the biomechanics of natural predator-prey interactions between rattlesnakes and their prey. In fact, studies that quantify the dynamics of prey capture by vertebrates in the wild are rare, often tracking the predator via accelerometers or animal-borne cameras (e.g. cheetahs^11^, birds^34^, and whales^35^). Although these are extremely valuable approaches, they often cannot record the critical movements of the prey item during the interaction. Alternatively, some situations facilitate the recording of an event with a specified volume (e.g. fishes^36^,^37^), although these often focus only on the predator or the prey, not both. Due to the predictability of the location of the predator strike, the sit-and-wait predation of rattlesnakes permits the tracking of both predator and prey within a specified volume.

These initial recordings revealed that the factors that dictate the outcome of the strike were variable. Kangaroo rat position and behavior at the initiation of the snake strike were variable. Two misses were due to an error on the part of the rattlesnake, where the snake initiated the strike too late and the kangaroo rat had passed by the time the jaws of the snake reached the location (Supplementary Video S1). This is not surprising given that past studies on rattlesnakes have also noted the difficulty involved in successfully striking moving prey^30^,^38^,^39^. One successful strike occurred with the kangaroo rat only 4.8 cm from the snake. There was no escape response despite facing the snake. Thus, it is likely that a certain distance is necessary to provide enough time to detect the snake and execute an evasive maneuver.

A recent study examined defensive strikes from both vipers (*Agkistrodon piscivorus* and *C. atrox*) and non-venomous ratsnakes (*Pantherophis obsoletus*), and found that strike performance was not different between them^29^. Our results, although variable, indicate that rattlesnakes (at least *C. scutulatus*) have the ability to greatly exceed the defensive strike speeds and accelerations observed in laboratory studies: The maximum velocities achieved during successful (4.2 ms^−1^) and unsuccessful (4.8 ms^−1^) strikes were 19% and 36% faster, respectively, than the maximum values observed by ref. 29. Similarly, our average maximum value of acceleration across all trials was 362 ms^−2^, 30% greater than the maximum observed by ref. 29. Another recent laboratory study examined the defensive and predatory strikes of *C. atrox*, and observed maximum acceleration (878 ms^−2^) and velocity (5.5 ms^−1^) values during predatory strikes that exceeded those of our study^27^. This might simply be due to differences between species, differences in body size, or simply different levels of motivation between studies. We lack data for *C. atrox* strikes in nature, so it is unclear whether the laboratory trials represent comparable values to snakes in nature. Regardless, we argue that strikes of snakes in nature must be quantified before any definitive conclusion concerning relative performance is made. There are a number of reasons to expect higher (and more variable) performance in nature, including lack of stress, elevated motivation, natural light levels and temperature, and natural prey behavior.

The escape response and behavior of the kangaroo rats were equally impressive, and we provide some of the first data regarding escape performance of small mammals during natural interactions. The average response time, from the onset of snake movement to the first observable motion of the kangaroo rat, was 61.5 ± 10.6 ms, which is at the lower end of the mammalian startle response^40^. Thus, the performance of the prey in our study can be considered extremely high. Compared to Pacific jumping mice (*Zapus trinotatus*)^41^, the *D. merriami* in our study were larger and reached velocities that were almost 50% greater (Table 1). Given that *Z. trinotatus* is assumed to amplify power by storing elastic energy in their distal tendons, and that we found higher levels of performance in the kangaroo rats in our study, we predict that the kangaroo rats are likely exhibiting power amplification via elastic energy storage. This is in contrast to steady locomotion in kangaroo rats, which does not involve elastic energy storage^42^.

We propose that the rattlesnake-kangaroo rat system is a model system for studying the dynamics of high-power predator-prey interactions, given that they can be observed (with some effort) under completely natural conditions. This system could be used to test a number of important questions about predation success and prey escape responses in nature. Given the extreme performance on the part of the snakes and kangaroo rats, it is very likely that elastic energy storage is important for both species in circumventing the limits of neuromuscular function. Future studies should address this possibility. Finally other species of rattlesnake (e.g. *C. cerastes*) consume other species of kangaroo rat (e.g. *D. deserti*)^43^, opening up the possibility of a comparative study across predators and prey.

## Methods

Six *C. scutulatus* (mass: 250 ± 39 g; snout-vent length: 69 ± 33 cm) were caught near Rodeo, New Mexico during the summer of 2015. Three were female and three were male. Snakes were anesthetized and implanted with a small, temperature-sensitive radiotransmitter as in ref. 30. Following complete recovery, snakes were released and tracked. Kangaroo rats (43 ± 4.2 g) were captured, weighed, and marked every night. We habituated kangaroo rats to forage for sterilized commercial birdseed by scattering seed in the vicinity of marked animals, and increased the probability of snake encounters by scattering seed in the vicinity of foraging snakes. When a snake was found in a predatory ambush coil in areas where we had marked kangaroo rats, two infrared-sensitive high-speed video cameras (Edgertronic) operating at 500 images s^−1^ were oriented towards the snake in order to capture three-dimensional motions. Following a strike attempt, a large calibration object with known dimensions encompassing the volume of the predator-prey interaction was placed in the field of view in order to obtain 3D coordinates. The fields of view were illuminated using multiple infrared flood lights (CMVision LED Infrared Illuminators), providing illumination for cameras to visualize the snake and kangaroo rat under natural ambient light conditions. Both successful and unsuccessful strikes were recorded.

Video files were converted from MOV to AVI and digitized in MATLAB 2013 using DLTDV5^44^. Points digitized included the (1) tip of the upper jaw, (2) tip of the lower jaw, (3) a point just posterior to the head of the snake, and (4) the base of the cranium of the kangaroo rat. Three-dimensional coordinates of points 3 and 4 were used to calculate displacement (change in position over time), instantaneous velocity (first derivative of displacement), and instantaneous acceleration (second derivative of displacement) of the snake and kangaroo rat. A quintic spline (in Igor Pro) was used to smooth the displacement data prior to calculating velocity and acceleration. The three-dimensional distance between the snake and kangaroo rat was determined throughout the strike and evasion. In addition, maximum gape and time to maximum gape (TTMG) of the snake were calculated. This work was approved by San Diego State University’s IACUC, and all methods were performed in accordance with the relevant guidelines and regulations put forth by IACUC.

## Additional Information

**How to cite this article**: Higham, T. E. *et al*. Rattlesnakes are extremely fast and variable when striking at kangaroo rats in nature: Three-dimensional high-speed kinematics at night. *Sci. Rep.* **7**, 40412; doi: 10.1038/srep40412 (2017).

**Publisher's note:** Springer Nature remains neutral with regard to jurisdictional claims in published maps and institutional affiliations.

## Supplementary Material

Supplementary Movie

Supplementary Information

## Figures and Tables

**Figure 1:**
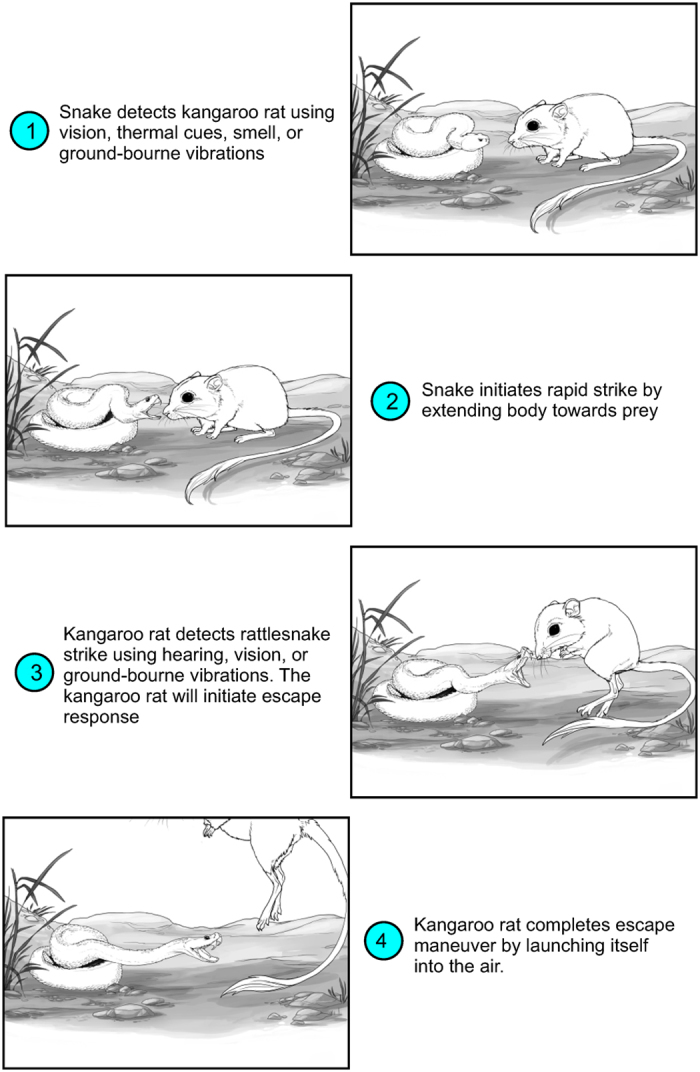
The predictive framework for escape maneuvers of kangaroo rats in response to strikes from rattlesnakes. This sequence of events is expected during natural interactions, and we observed this in multiple interactions. Amy Cheu provided these illustrations.

**Table 1:** Summary of rattlesnake and kangaroo rat performance. Values are average (min – max).

	Max snake velocity (m s^−1^)	Max snake acceleration (m s^−2^)	Max KR velocity (m s^−1^)	Max KR acceleration (m s^−2^)	Initial pred-prey distance (cm)	Time to capture (ms)	TTMG (ms)
Miss (N = 3)	3.1 (2.0–4.8)	170.8 (44.3–416.0)	3.4 (2.6–4.2)	612.4 (465.2–873.9)	16.9 (14.8–18.3)	—	43.3 (26–64)
Hit (N = 4)	3.5 (2.8–4.2)	506.1 (424.0–680.9)	3.3 (1.5–4.5)	619.7 (213.1–958.1)	12.9 (4.6–20.6)	105 48–170	35 (28–42)

KR, kangaroo rat; TTMG, time to maximumg ape.
