# Supplementary material for: Rattlesnakes are extremely fast and variable when striking at kangaroo rats in nature: Three-dimensional high-speed kinematics at night
Source: Sci Rep. 2017 Jan 13;7:40412. doi: 10.1038/srep40412 (PMC5234031; doi:10.1038/srep40412)
Supplement: Supplementary Information [file srep40412-s2.pdf]

Supplementary information for:

Rattlesnakes are extremely fast and variable when striking at kangaroo rats in nature: Three-dimensional high-speed kinematics at night

Timothy E. Higham[1]*, Rulon W. Clark[2]*, Clint E. Collins[1], Malachi D. Whitford[2], and Grace A. Freymiller[2].

[1]Department of Biology, University of California, Riverside, CA 92521, USA
[2]Department of Biology, San Diego State University, San Diego, CA 92182, USA

Video S1. This video shows a high-speed strike from a Mohave rattlesnake on a kangaroo rat. This was an unsuccessful attempt. The video was recorded at 500 frames per second, but is slowed down to 30 frames per second in this video.
